# Anaemia is associated with higher disease activity in axial spondyloarthritis but is not an independent predictor of spinal radiographic progression: data from the Swiss Clinical Quality Management Registry

**DOI:** 10.1007/s10067-023-06662-0

**Published:** 2023-06-08

**Authors:** Raphael Micheroli, Seraphina Kissling, Kristina Bürki, Burkhard Möller, Axel Finckh, Michael J. Nissen, Pascale Exer, René Bräm, Diego Kyburz, Andrea Rubbert-Roth, Michael Andor, Xenofon Baraliakos, Manouk de Hooge, Oliver Distler, Almut Scherer, Adrian Ciurea

**Affiliations:** 1 Department of Rheumatology, Zurich University Hospital, University of Zurich, Gloriastrasse 25, CH-8091 Zurich, Switzerland; 2 Swiss Clinical Quality Management Foundation, Zurich, Switzerland; 3 Department of Rheumatology and Immunology, Inselspital, Bern, Switzerland; 4 Department of Rheumatology, Geneva University Hospital, Geneva, Switzerland; 5 Gemeinschaftspraxis Rheuma-Basel, Basel, Switzerland; 6 Swiss Ankylosing Spondylitis Association, Zurich, Switzerland; 7 Department of Rheumatology, University Hospital Basel, University of Basel, Basel, Switzerland; 8 Deparment of Rheumatology, Cantonal Hospital St, Gallen, St. Gallen, Switzerland; 9 Rheumatologie Im Zürcher Oberland, Uster, Switzerland; 10 Rheumazentrum Ruhrgebiet Herne, Ruhr-University Bochum, Bochum, Germany; 11 Department of Rheumatology, Ghent University Hospital, Ghent, Belgium

**Keywords:** **A**naemia, Ankylosing spondylitis, Axial spondyloarthritis, Biomarker, Radiographic progression

## Abstract

**Objective:**

As anaemia represents a biomarker for increased radiographic damage in rheumatoid arthritis, we aimed to investigate whether it independently predicts spinal radiographic progression in axial spondyloarthritis (axSpA).

**Methods:**

AxSpA patients with available haemoglobin levels from the prospective Swiss Clinical Quality Management Registry were included for comparison of patients with and without anaemia. Spinal radiographic progression was assessed according to the modified Stoke Ankylosing Spondylitis Spinal Score (mSASSS) in patients with ankylosing spondylitis (AS) if ≥ 2 sets of spinal radiographs were available every 2 years. The relationship between anaemia and progression (defined as an increase ≥ 2 mSASSS units in 2 years) was analysed with generalized estimating equation models after adjustment for the Ankylosing Spondylitis Disease Activity Score (ASDAS) and potential confounding, as well as after multiple imputations of missing values.

**Results:**

A total of 212/2522 axSpA patients presented with anaemia (9%). Anaemic patients had higher clinical disease activity, higher acute phase reactants and more severe impairments in physical function, mobility and quality of life. In the subgroup of patients with AS (*N* = 433), a comparable mSASSS progression was found in anaemic and non-anaemic patients (OR 0.69, 95% CI 0.25 to 1.96, *p* = 0.49). Age, male sex, baseline radiographic damage and ASDAS were associated with enhanced progression. The results were confirmed in complete case analyses and with progression defined as the formation of ≥ 1 syndesmophyte in 2 years.

**Conclusion:**

Although anaemia was associated with higher disease activity in axSpA, it did not additionally contribute to the prediction of spinal radiographic progression.

**Table:** 

**Key Points** • *Anaemia is associated with higher disease activity and more severely impaired physical function, mobility and quality of life in axSpA.* • *Anaemia does not provide an additional value to ASDAS for prediction of spinal radiographic progression.*

## Introduction

Spinal structural damage and its progression are major determinants of functional impairment in patients with axial spondyloarthritis (axSpA) and particularly in patients with ankylosing spondylitis (AS) [1, 2]. Structural damage is limited to a selected group of patients, and it is the most conclusive predictor of further progression [3, 4]. Only a few additional predictors of progression have been identified over the last years, including acute phase reactants, fatty post-inflammatory vertebral corner changes on magnetic resonance imaging (MRI), male gender, smoking and manual jobs [5]. The level of disease activity as assessed by the Ankylosing Spondylitis Disease Activity Score (ASDAS) proved to be a better biomarker of progression when compared to some of its components, in particular the Bath Ankylosing Disease Activity Index (BASDAI) and the level of C-reactive protein (CRP) [6, 7]. In contrast, treatment with tumour necrosis factor inhibitors (TNFi) seems to be able to retard radiographic progression [8]. A recent analysis suggested that a genetic factor, the ryanodine receptor 3 gene, might be associated with severe radiographic damage [9]. Other biomarkers proved of modest benefit [10–12]. Anaemia predicted radiographic progression independently of common disease activity parameters in rheumatoid arthritis (RA) [13, 14]. As anaemia is known to be associated with disease activity in AS [15], we thought to determine whether it could provide an additional predictive value to ASDAS in AS.

## Methods

### Study population

We took advantage of the Swiss Clinical Quality Management (SCQM) registry of patients with axSpA, as diagnosed by a board-certified rheumatologist in Switzerland [16]. The registry was initiated in 2005. Clinical assessments were performed according to the recommendations of the Assessment of SpondyloArthritis International Society (ASAS) [17]. Patients were included in the current study if, in addition to diagnosis, they also fulfilled the ASAS classification criteria for axSpA [18] and if an assessment of haemoglobin (Hb) was available (either at inclusion or at the start of a radiographic interval, depending on the analysis performed; see below). For the analysis of spinal radiographic progression, only patients fulfilling the radiographic criterion of the modified New York classification [19] were considered, as patients with nonradiographic disease status have only minimal spinal progression [20]. Written informed consent was obtained from all patients. The study was approved by the Ethics Commission of the Canton of Zurich (KEK-ZH-Nr. 2014–0439). We have chosen the database snapshot of August 1, 2016, for the following reasons: (a) it represented the time-point of radiographic scoring of available spinal radiographs in SCQM; (b) no biological or targeted-synthetic disease-modifying drugs other than TNFi had been approved at this time-point to additionally influence our analyses.

### Laboratory assessments

Anaemia was defined according to the definition of the World Health Organization (WHO): Hb level below 12 g/dl in women and below 13 g/dl in men [21]. Hb concentrations, C-reactive protein (CRP) levels and erythrocyte sedimentation rates (ESR) were obtained from certified local laboratories and their levels were entered in the database by the treating rheumatologist.

### Radiographic assessments

As data on radiographic progression in axSpA is limited, SCQM rheumatologists were instructed to perform radiographs of the pelvis and of the spine every 2 years if clinically appropriate and in the absence of contraindications. Images were uploaded to the SCQM database. Sacroiliac joint (SIJ) damage was assessed on anteroposterior pelvis radiographs according to the radiographic criterion of the modified New York classification criteria [19] centrally by two calibrated readers of the SCQM scientific committee. Paired reading of lateral radiographs of the cervical and lumbar spine taken every 2 years (up to 5 radiographic intervals, corresponding to 10 years of follow-up) was performed by two calibrated readers according to the modified Stoke Ankylosing Spondylitis Spine Score (mSASSS) [22] as already published (radiographs were not scored again) [7]. Mean scores per vertebral corner (VC) were used. Images were disregarded in the presence of > 3 missing VCs per cervical and lumbar segment [7]. Independent adjudication was performed by a third scorer if the mSASSS status scores varied by ≥ 5 mSASSS units. Radiographic progression was defined either as an increase in mSASSS of at least 2 units in 2 years or as the formation of at least one syndesmophyte in 2 years, with syndesmophytes only counted if both readers agreed on their presence.

### Statistical analysis

Comparisons between characteristics of patients with and without anaemia were performed using Fisher’s exact test for nominal variables and the Mann–Whitney test for continuous variables. Generalized estimating equation (GEE) models with an “exchangeable” correlation structure were used to examine the relationship between anaemia and radiographic progression over time [7]. The progression of at least 2 mSASSS units in 2 years was modelled with the use of the binomial family and the logistic link function, and the models were adjusted for the following variables: age, sex, ASDAS, either mSASSS at the start of the interval or the presence of syndesmophytes at the start of the interval, prior treatment with TNFi, current smoking status and the respective length of the radiographic interval. Multiple imputation was used for missing covariate data, with ASDAS derived by passive imputation (missingness: ASDAS 104/617 intervals, smoking status 84/617 intervals, anaemia 70/617 intervals). A Wald test was performed to assess the importance of the addition of anaemia in the prediction model for progression.

## Results

### Characteristics of axSpA patients with respect to anaemia status

**Fig. 1:**
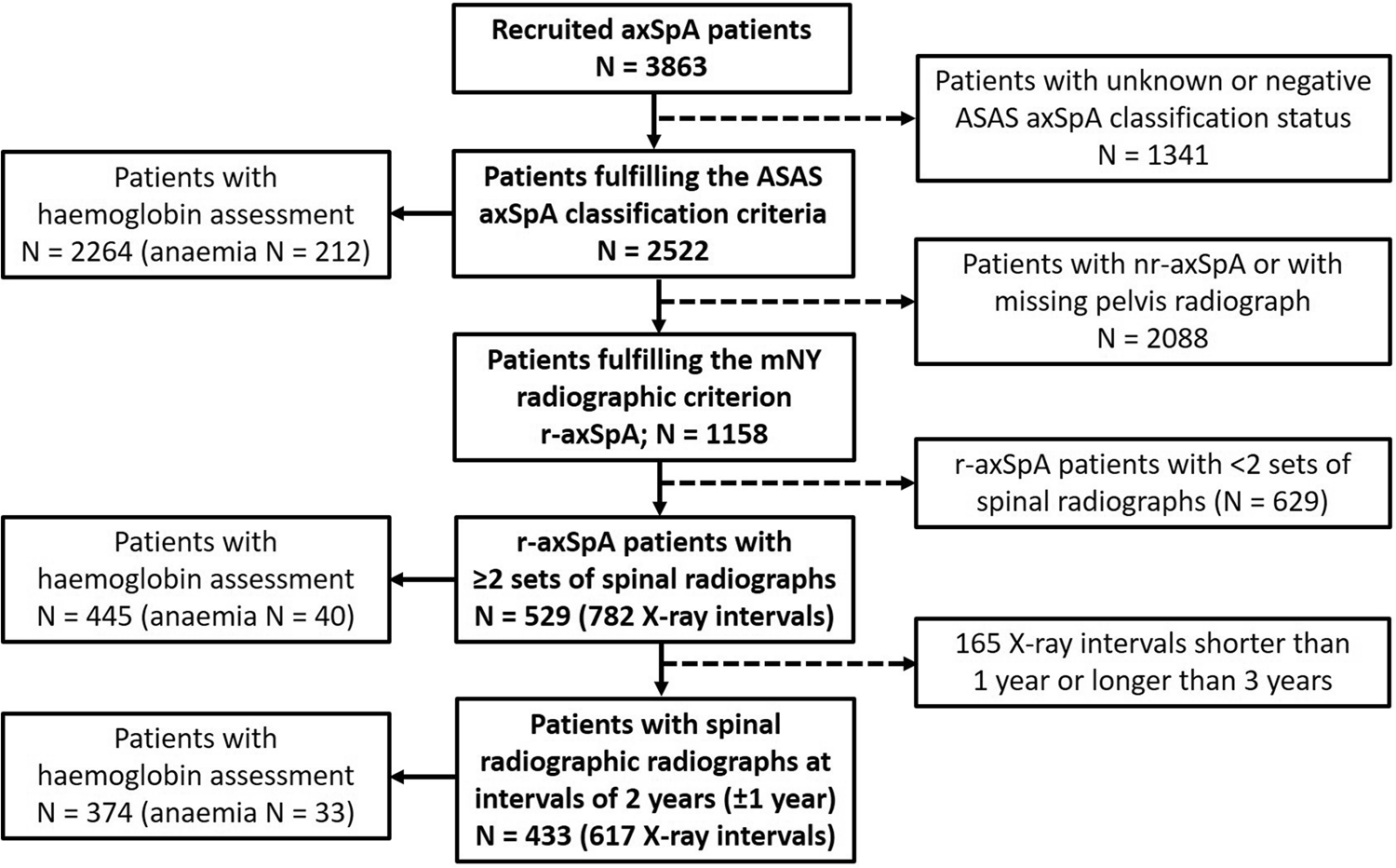
Disposition of patients with axial spondyloarthritis in the Swiss Clinical Quality Management (SCQM) registry. axSpA, axial spondyloarthritis; ASAS, Assessment in SpondyloArthritis International Society; mNY, modified New York classification; nr-axSpA, nonradiographic axial spondyloarthritis; r-axSpA, radiographic axial spondyloarthritis

The disposition of patients at inclusion in the SCQM axSpA cohort is depicted in a flow chart in Fig. 1. Out of 3863 with a clinical diagnosis of axSpA, 2522 fulfilled the ASAS axSpA classification criteria. Hb levels were available in 2264 patients (89.8%). Anaemia was observed in 212 of these patients (9.2%). Characteristics of patients with normal Hb levels versus patients with anaemia are shown in Table 1. A higher proportion of patients with anaemia were of female sex (51% vs. 39% in non-anaemic patients). Anaemic patients were slightly older and had a longer disease duration. While the proportion of HLA-B27 positivity was comparable between the two groups, the percentage of patients with definite radiographic SIJ changes was higher in patients with anaemia (80% vs. 72%). Patients with anaemia had significantly higher disease activity as assessed by subjective means (BASDAI) and objective parameters (acute phase reactants, proportion of patients with peripheral arthritis and hip arthritis). In contrast, enthesitis and dactylitis were similarly distributed between the two groups. Paralleling disease activity, physical function, mobility and quality of life (as assessed by the Bath Ankylosing Spondylitis Functional and Mobility Indices and the European Quality of Life Questionnaire 5 domains (EQ-5D), respectively) were more severely impaired in patients with anaemia (Table 1). A higher percentage of non-anaemic patients were already treated with TNFi at inclusion in the SCQM axSpA cohort (23% vs. 16% in anaemic patients), while more patients with anaemia were treated with conventional synthetic disease-modifying anti-rheumatic drugs (19% vs. 12%).

**Table 1:** Characteristics of patients at inclusion in SCQM

Parameter	*N* 2264	Non-anaemic *N* = 2052	Anaemic *N* = 212	*p*
Haemoglobin, G/l	**2264**	**14.3 (1.2)**	**11.7 (1.0)**	** < 0.001**
Male sex, *N* (%)	**2264**	**1256 (61.2)**	**103 (48.6)**	** < 0.001**
Age, years	2264	39.1 (11.3)	41.0 (13.3)	0.16
Symptom duration, years	**2209**	**12.7 (11.1)**	**14.3 (11.6)**	**0.05**
Radiographic axSpA, *N* (%)	**1508**	**979 (71.8)**	**115 (79.9)**	**0.04**
HLA-B27, *N* (%)	2075	1472 (78.4)	162 (82.2)	0.23
BASDAI	**1925**	**4.7 (2.3)**	**5.2 (2.2)**	**0.01**
ESR	**2169**	**13.8 (13.5)**	**37.6 (25.0)**	** < 0.001**
CRP (mg/l)	**2208**	**9.5 (13.7)**	**29.4 (31.4)**	** < 0.001**
Elevated CRP, *N* (%)	**2193**	**720 (36.2)**	**151 (73.0)**	** < 0.001**
BASFI	**1940**	**3.3 (2.6)**	**4.1 (2.7)**	** < 0.001**
BASMI	**2139**	**2.0 (1.9)**	**2.8 (2.3)**	** < 0.001**
EQ-5D	**1909**	**61.3 (22.0)**	**54.8 (22.2)**	** < 0.001**
Current arthritis, *N* (%)	**2226**	**624 (30.9)**	**97 (46.6)**	** < 0.001**
Current hip arthritis, *N* (%)	**2206**	**250 (12.5)**	**40 (19.3)**	**0.01**
Current enthesitis, *N* (%)	2224	1327 (65.8)	142 (68.9)	0.40
Current dactylitis, *N* (%)	2226	77 (3.8)	7 (3.4)	0.85
Current smoking, *N* (%)	**1905**	**658 (38.1)**	**51 (29.4)**	**0.02**
Body mass index	**2070**	**25.4 (4.5)**	**24.3 (4.7)**	** < 0.001**
csDMARD use, *N* (%)	**2264**	**240 (11.7)**	**39 (18.6)**	**0.01**
TNFi use, *N* (%)	**2264**	**480 (23.4)**	**33 (15.6)**	** < 0.001**

Except where indicated otherwise, values are the mean (SD). Statistically significant differences between groups are shown in bold. Patients with available haemoglobin level at inclusion in SCQM. *axSpA*, axial spondyloarthritis; *BASDAI*, Bath Ankylosing Spondylitis Disease Activity Index; *BASFI*, Bath Ankylosing Spondylitis Functional Index; *BASMI*, Bath Ankylosing Spondylitis Metrology Index; *CRP*, C-reactive protein; *csDMARD*, conventional synthetic disease-modifying anti-rheumatic drug; *ESR*, Erythrocyte Sedimentation Rate; *EQ-5D*, European Quality of Life 5-domains; *HLA-B27*, human leucocyte antigen B27; *IBD* = inflammatory bowel disease; *TNFi*, tumour necrosis factor inhibitor

### Characteristics of AS patients with respect to anaemia status

Spinal radiographic progression was assessed in patients with radiographic disease, as spinal progression is very limited in patients with nonradiographic axSpA [17]. Hb assessments were available in 445 patients out of 529 patients with at least two sets of spinal radiographs (84.1%) and in 374/433 patients with radiographic intervals at 2-year intervals (86.4%) (Fig. 1). Anaemia was present in 9% of patients in the group with at least 2 sets of radiographs, as well as in the smaller group of patients with sets of radiographs at intervals of 2 years. Comparison of characteristics of non-anaemic vs. anaemic r-axSpA patients is shown for both groups of patients with sequential radiographs in Table 2. The proportion of patients with different numbers of radiographic intervals was comparable in both groups. In line with the results found at inclusion in the SCQM cohort for all axSpA patients, anaemic r-axSpA patients had higher disease activity and more restricted physical function, spinal mobility and quality of life, although numerical differences did not always reach statistical significance given the lower number of patients in the respective comparison groups. The most important differences between anaemic and non-anaemic patients were recorded for acute phase reactants (number of patients with elevated CRP, as well as the height of the CRP and the ESR elevation) (Table 2).

**Table 2:** Characteristics of r-axSpA patients with ≥ 2 sets of spinal radiographs at the time-point of their first radiograph

	A. All patients with ≥ 2 sets of spinal radiographs						B. Patients with ≥ 2 sets of spinal radiographs at intervals of 2 years					
	All patients		Patients with available haemoglobin levels				All patients		Patients with available haemoglobin levels			
Parameter	*N*	*N* = 529	*N*	Non-anaemic *N* = 405	Anaemic *N* = 40	*p*	*N*	*N* = 433	*N*	Non-anaemic *N* = 341	Anaemic *N* = 33	*p*
Haemoglobin, G/l	445	14.2 (1.4)	445	14.4 (1.2)	11.9 (0.8)	< 0.001	374	14.2 (1.4)	374	14.4 (1.2)	11.9 (0.7)	< 0.001
Male sex, *N* (%)	529	354 (66.9)	445	280 (69.1)	25 (62.5)	0.38	433	285 (65.8)	374	231 (67.7)	21 (63.6)	0.70
Age, years	529	39.7 (11.1)	445	39.5 (11.0)	40.6 (11.6)	0.55	433	40.3 (11.0)	374	39.9 (11.0)	42.8 (11.1)	0.14
Symptom duration, years	520	13.7 (9.8)	**441**	**13.3 (9.7)**	**15.8 (8.8)**	**0.04**	425	13.8 (9.8)	**370**	**13.3 (9.6)**	**16.3 (8.9)**	**0.03**
HLA-B27, *N* (%)	480	389 (81.0)	401	299 (81.7)	30 (85.7)	0.65	392	316 (80.6)	337	249 (80.8)	25 (86.2)	0.62
BASDAI	440	4.3 (2.3)	425	4.3 (2.3)	4.8 (2.3)	0.14	369	4.2 (2.3)	357	4.2 (2.3)	5.0 (2.0)	0.06
ESR	440	16.3 (16.2)	**438**	**14.2 (14.0)**	**37.1 (22.0)**	** < 0.001**	368	15.6 (14.9)	**367**	**13.8 (13.1)**	**34.7 (18.5)**	** < 0.001**
ASDAS-CRP	442	2.5 (1.1)	**420**	**2.5 (1.1)**	**3.3 (1.3)**	** < 0.001**	351	2.8 (1.1)	**350**	**2.8 (1.1)**	**3.4 (1.1)**	**0.003**
CRP (mg/l), median, IQR	437	8 (1.2; 3.8)	**435**	**7 (3.0; 11.0)**	**15 (8.5; 38)**	** < 0.001**	365	8 (3; 11)	**364**	**7 (3; 10)**	**14.5 (8; 27)**	** < 0.001**
Elevated CRP, *N* (%)	436	186 (42.7)	**434**	**157 (39.8)**	**29 (74.4)**	** < 0.001**	364	147 (40.4)	**363**	**124 (37.5)**	**23 (71.9)**	** < 0.001**
BASFI	445	3.1 (2.6)	430	3.1 (2.5)	3.6 (2.8)	0.29	373	3.1 (2.6)	361	3.0 (2.6)	3.6 (2.5)	0.16
BASMI	452	2.2 (2.0)	**438**	**2.1 (1.9)**	**2.9 (2.3)**	**0.05**	375	2.2 (2.0)	366	2.1 (1.9)	2.8 (2.3)	0.09
mSASSS	529	6.5 (12.4)	445	6.8 (12.7)	5.1 (9.4)	0.62	433	6.6 (12.5)	374	6.8 (12.8)	6.2 (10.1)	0.59
Syndesmophytes, *N* (%)	529	180 (34.0)	445	142 (35.1)	11 (27.5)	0.39	433	148 (34.2)	374	118 (34.6)	12 (36.4)	0.85
EQ-5D	440	64.8 (21.2)	425	65.1 (21.4)	61.1 (20.1)	0.15	370	65.1 (21.6)	358	65.6 (21.8)	60.3 (19.0)	0.06
Current arthritis, *N* (%)	453	144 (31.8)	**441**	**118 (29.4)**	**21 (52.5)**	**0.004**	378	108 (28.6)	**370**	**89 (26.4)**	**16 (48.5)**	**0.01**
Current hip arthritis, *N* (%)	449	59 (13.1)	**436**	**48 (12.1)**	**11 (27.5)**	**0.01**	372	41 (11.0)	363	34 (10.3)	7 (21.2)	0.08
Current enthesitis, *N* (%)	456	254 (55.7)	**443**	**218 (54.1)**	**29 (72.5)**	**0.03**	381	207 (54.3)	**372**	**178 (52.5)**	**24 (72.7)**	**0.03**
Current dactylitis, *N* (%)	449	8 (1.8)	**436**	**5 (1.3)**	**3 (7.5)**	**0.03**	372	5 (1.3)	363	3 (0.9)	2 (6.1)	0.07
Current smoking, *N* (%)	436	171 (39.2)	**421**	**156 (40.6)**	**8 (21.6)**	**0.03**	366	140 (38.2)	**354**	**128 (39.6)**	**5 (16.1)**	**0.01**
Body mass index	449	25.1 (4.1)	**435**	**25.2 (4.1)**	**23.5 (4.0)**	**0.01**	373	25.2 (4.3)	**364**	**25.3 (4.3)**	**23.7 (3.4)**	**0.03**
csDMARD use, *N* (%)	529	89 (16.8)	445	70 (17.3)	5 (12.5)	0.52	433	70 (16.2)	374	56 (16.4)	3 (9.1)	0.33
TNFi use, *N* (%)	529	181 (34.2)	**445**	**147 (36.3)**	**8 (20.0)**	**0.05**	433	163 (37.6)	**374**	**134 (39.3)**	**6 (18.2)**	**0.02**
Nb. of X-ray intervals, *N* (%)	529		445			0.08	433		374			0.30
• 1		339 (64.1)		256 (63.2)	18 (45.0)			294 (67.9)		231 (67.7)	28 (54.5)	
• 2		126 (23.8)		102 (25.2)	13 (32.5)			92 (21.2)		72 (21.4)	11 (33.3)	
• 3		48 (9.1)		34 (8.4)	8 (20.0)			35 (8.1)		26 (7.6)	4 (12.1)	
• 4		15 (2.8)		12 (3.0)	1 (2.5)			11 (2.5)		10 (2.9)	0 (0.0)	
• 5		1 (0.2)		1 (0.2)	0 (0.0)			1 (0.2)		1 (0.3)	0 (0.0)	

Except where indicated otherwise, values are the mean (SD). Statistically significant differences between groups are shown in bold. *ASDAS*, Ankylosing Spondylitis Disease Activity Score; *BASDAI*, Bath Ankylosing Spondylitis Disease Activity Index; *BASFI*, Bath Ankylosing Spondylitis Functional Index; *BASMI*, Bath Ankylosing Spondylitis Metrology Index; *CRP*, C-reactive protein levels; *csDMARD*, conventional synthetic disease-modifying anti-rheumatic drug; *ESR*, Erythrocyte Sedimentation Rate; *EQ-5D*, European Quality of Life 5-domains; *HLA-B27*, human leucocyte antigen B27; *mSASSS*, modified Stoke Ankylosing Spondylitis Spinal Score; *Nb*, number; *NSAIDs*, nonsteroidal anti-inflammatory drugs; *TNFi*, tumour necrosis factor inhibitor

### Crude spinal progression analyses

**Fig. 2:**
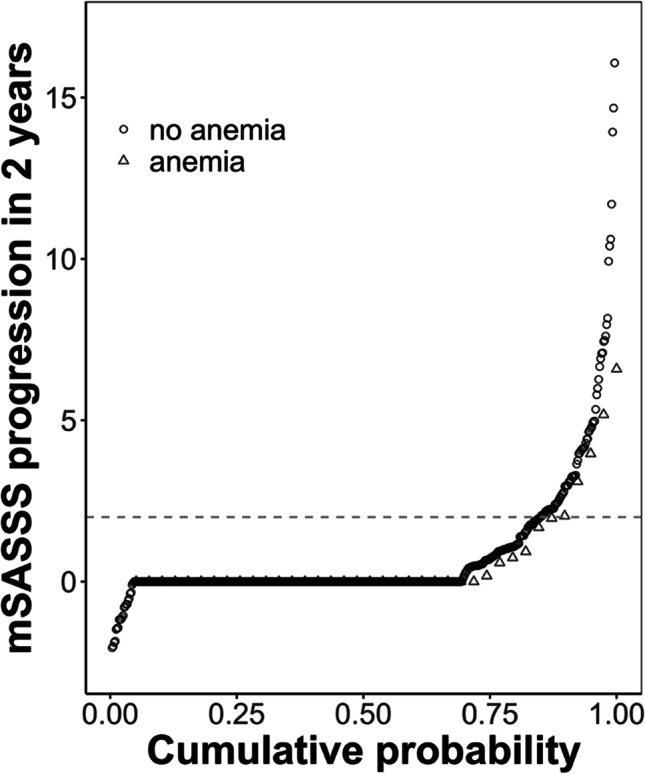
Cumulative probability plot depicting 2-year progression in the modified Stoke Ankylosing Spondylitis Spinal Score (mSASSS). The change in mSASSS values from start to end of individual 2-year radiographic intervals is shown for patients with anaemia (triangles) and for patients without anaemia (circles)

The crude odds of spinal radiographic progression in anaemic versus non-anaemic patients were comparable (OR 1.01, 95% confidence interval (CI) 0.45; 2.23, *p* = 0.99). This result is also depicted as a cumulative probability plot for mSASSS progression over 2 years for individual radiographic intervals in patients with and without anaemia in Fig. 2.

### Adjusted spinal progression analyses

Anaemia was not an independent predictor of spinal progression after adjustment for sex, baseline damage, ASDAS, smoking, TNFi treatment, age and length of the radiographic interval, and multiple imputations of missing covariate data (OR 0.69, 95% CI 0.25 to 1.96, *p* = 0.49; Table 3, part A). Age, male sex, baseline mSASSS and ASDAS were associated with accelerated progression in this model. We found no evidence that adding anaemia to the progression model significantly improved the model (Chi-squared value of 0.22, *p* = 0.64). The main results were confirmed in a complete case analysis (Table 3, part B). Moreover, there was no association of anaemia with spinal radiographic progression in the analyses performed with an alternative definition of progression, the formation of at least one syndesmophyte during an X-ray interval (OR 0.61, 95% CI 0.22 to 1.70, *p* = 0.35; Table 4).

**Table 3:** Multivariable analysis for the identification of factors associated with spinal radiographic progression in r-axSpA (progression defined as an increase in ≥ 2 mSASSS units in 2 years)

	A. Multiple imputation of missing data			B. Complete case analysis		
Variable	OR	95% CI	*p* value	OR	95% CI	*p* value
Anaemia	0.69	0.25; 1.96	0.49	0.77	0.26; 2.29	0.64
Male sex	**2.70**	**1.39; 5.23**	**0.003**	**2.93**	**1.38; 6.22**	**0.01**
mSASSS at start of each radiographic interval	**1.06**	**1.04; 1.08**	** < 0.001**	**1.06**	**1.04; 1.08**	** < 0.001**
ASDAS	**1.36**	**1.03; 1.80**	**0.03**	1.31	0.99; 1.74	0.06
Current smoking	1.10	0.60; 1.99	0.76	1.06	0.57; 1.98	0.86
TNFi use prior to radiographic interval	0.67	0.40; 1.14	0.14	0.84	0.49; 1.44	0.52
Age (5 years increments)	**1.26**	**1.11; 1.43**	** < 0.001**	**1.26**	**1.09; 1.45**	**0.002**
Length of the radiographic interval	1.84	0.94; 3.61	0.08	1.56	0.72; 3.38	0.26

Analyses performed in 617 radiographic intervals from 433 patients (104 events) in A, and in 500 radiographic intervals from 361 patients (90 events) in B

Statistically significant results are shown in bold

*ASDAS*, Ankylosing Spondylitis Disease Activity Score; *mSASSS*, Modified Stoke Ankylosing Spondylitis Spinal Score; *r-axSpA*, radiographic axial spondyloarthritis; *TNFi*, tumour necrosis factor inhibitor

**Table 4:** Multivariable analysis for the identification of factors associated with spinal radiographic progression in r-axSpA (progression defined as the formation of ≥ 1 syndesmophyte in 2 years)

	A. multiple imputation of missing data			B. Complete case analysis		
Variable	OR	95% CI	*p* value	OR	95% CI	*p* value
Anaemia	0.61	0.22; 1.70	0.35	0.79	0.27; 2.30	0.67
Male sex	1.76	0.86; 3.60	0.12	**2.33**	**1.06; 5.12**	**0.04**
Presence of syndesmophytes at start of each radiographic interval	**7.85**	**4.14; 14.9**	** < 0.001**	**6.72**	**3.51; 12.9**	** < 0.001**
ASDAS	1.26	0.97; 1.65	0.08	1.22	0.93; 1.59	0.15
Current smoking	0.87	0.50; 1.52	0.64	0.80	0.44; 1.43	0.45
TNFi use prior to radiographic interval	0.61	0.36; 1.02	0.06	0.76	0.44; 1.29	0.31
Age (5 years increments)	**1.17**	**1.04; 1.31**	**0.01**	**1.16**	**1.02; 1.32**	**0.02**
Length of the radiographic interval	**1.95**	**1.01; 3.77**	**0.05**	1.65	0.80; 3.41	0.18

Analyses performed in 617 radiographic intervals from 433 patients (104 events) in A, and in 500 radiographic intervals from 361 patients (88 events) in B

Statistically significant results are shown in bold

*ASDAS*, Ankylosing Spondylitis Disease Activity Score; *r-axSpA*, radiographic axial spondyloarthritis; *TNFi*, tumour necrosis factor inhibitor

## Discussion

Anaemia, as defined by the WHO, was detectable in 9% of patients with axSpA in our cohort. This proportion is two times higher than the one found in patients with recently diagnosed, incident AS in a recent publication [23]. However, our cohort includes patients with longstanding disease with > 10 years of mean symptom duration. The frequency of anaemia is lower than in an Italian analysis (15%) of patients requiring biologic treatment [15]. In that particular study, its pathogenesis was established as anaemia of inflammation, characterized by normal mean corpuscular volume of erythrocytes, low serum iron levels and iron-binding capacity, and elevated serum ferritin, after exclusion of other causes of anaemia. It resolved in 82% of patients following treatment with a TNFi [15], a finding confirmed by other studies in AS [24, 25]. The proportion of patients already treated with TNFi at recruitment in our cohort was 23% in the non-anaemic group, contrasting with a lower percentage in the group of patients with anaemia (16%). Anaemia was still associated with higher BASDAI levels, more peripheral arthritis and particularly with a relevantly higher proportion of patients with elevated CRP and with higher levels of CRP as well as of ESR in both axSpA and AS.

Our group reported that anaemia is associated with a more severe progression of erosive damage in the SCQM cohort of patients with RA [13], a result that was confirmed in another RA population [14]. Follow-up analyses revealed that clinical disease activity was more closely associated with haemoglobin levels than with the effects of treatment with TNFi and interleukin-6 receptor inhibitors [26]. These findings formed the background for the analysis of radiographic progression in AS performed here. Disease activity, particularly when assessed by the ASDAS, is associated with the accelerated progression of osteoproliferative structural changes in AS [6]. The latter can be retarded by treatment with TNFi [7], as confirmed in additional observational analyses (reviewed in [8, 27]).

Our study reveals that, in contrast to RA, anaemia is of no additional benefit to common disease activity assessments for the prediction of radiographic progression in AS. We have used state-of-the-art statistical methods (GEE, multiple imputation of missing covariate data) and have adjusted our analyses for known predictors of progression and for time-varying treatment with TNFi. To simplify the therapeutic context, we have used data from the SCQM cohort at a time, when alternative biologic or targeted-synthetic drugs were not approved for axSpA. Complete case analyses confirmed the robustness of our results. MRI data on inflammatory or post-inflammatory SIJ or spinal changes is currently not available in SCQM, which represents a limitation of our analyses. The relationship between MRI spinal inflammation and new bone formation is quite complex, as reliable detection requires periods of at least 2 years’ duration and involves the intermediate development of fatty degeneration [28, 29]. Besides differences in what constitutes structural damage between RA and axSpA (predominantly erosive changes vs. osteoproliferation), the lower frequency and severity of anaemia in axSpA in comparison to RA [13, 14, 23, 25] might preclude a relevant impact on radiographic progression in axSpA. Indeed, our study may potentially be underpowered to detect small effects on radiographic damage progression associated with anaemia. Our results parallel a series of negative findings in the search for biomarkers for radiographic progression in axSpA [30]. Moreover, the added benefit of several serum markers proved to be very modest [10–12]. Applying new technologies to identify biomarkers, therefore, remains warranted [9, 30].

## Conclusion

Anaemia was associated with increased disease activity in axSpA, but in contrast to RA, it seemed to not independently predict radiographic structural damage.

## Data Availability

Restrictions apply to the availability of these data. Data is owned by a third party, the Swiss Clinical Quality Management in Rheumatic Diseases (SCQM) foundation. Data may be obtained after approval and permission from the licence holder (SCQM). Contact information for data request: scqm@hin.ch.
